# The Advances and Biomedical Applications of Imageable Nanomaterials

**DOI:** 10.3389/fbioe.2022.914105

**Published:** 2022-07-05

**Authors:** Xiaohong Xiang, Doudou Shi, Jianbo Gao

**Affiliations:** ^1^ Department of Radiology, The First Affiliated Hospital of Zhengzhou University, Zhengzhou, China; ^2^ Department of Gastroenterology, The Affiliated Hospital of Yan’an University, Yan’an, China

**Keywords:** imageable, nanomaterials, advantages, biomedical applications, characteristic

## Abstract

Nanomedicine shows great potential in screening, diagnosing and treating diseases. However, given the limitations of current technology, detection of some smaller lesions and drugs’ dynamic monitoring still need to be improved. With the advancement of nanotechnology, researchers have produced various nanomaterials with imaging capabilities which have shown great potential in biomedical research. Here, we summarized the researches based on the characteristics of imageable nanomaterials, highlighted the advantages and biomedical applications of imageable nanomaterials in the diagnosis and treatment of diseases, and discussed current challenges and prospects.

## 1 Introduction

The diagnosis and efficacy evaluation of human diseases mainly relies on imaging technology and laboratory tests. Many diseases are not easily detected early and result in poor prognosis (Cencini et al., 2021). And for chronic diseases such as cancers, the therapeutic effects and absorption efficiency of drugs are also limited due to the influence of certain barriers in human body (Patel and Patel, 2017). Additionally, the special physical and chemical properties of certain drugs directly affect their absorption and efficacy (Xu et al., 2018). The current detection and prognosis of diseases are still unsatisfactory, so more effective detection and treatment strategies need to be further explored.

Common imaging detection methods mainly include positron emission tomography (PET), single-photon emission computed tomography (SPECT), computed tomography (CT), ultrasound, magnetic resonance imaging (MRI), optical imaging (OI) and photoacoustic (PA) imaging. These methods have the characteristics of displaying anatomical structures and/or functional imaging, such as providing blood vessel and tissue information when using contrast agents in CT detection. At present, imaging technology is mainly used for disease screening, early diagnosis and preliminary evaluation of curative effects. While every imaging modality has its advantages and disadvantages. For example, CT can provide anatomical information, but the sensitivity needs to be improved; ultrasound and optical imaging can provide non-invasive imaging, but are limited in depth; MRI can provide better soft tissue and brain function information, but the cost of the examination is high and examination time is long; PET has a higher sensitivity, but is limited to the types of diseases (Hu et al., 2014). How to realize the complementary advantages of these imaging technologies is the concentration on current research.

At present, nanomaterials are the focus of medical research. With the discovery of various characteristics of nanomaterials, the research on the role of nanomaterials in diseases has been gradually developed. Nanomaterials themselves have small molecular weights, good biocompatibility, and have the characteristics of ultrasound, optics and electromagnetics (Wong et al., 2020). In addition, nanomaterials can also be modified with fluorescence or special groups. These advantages make nanomaterials have great potential in drug delivery systems, medical imaging and diagnostic platforms, implantable materials, and tissue regeneration (Mabrouk et al., 2021). Therefore, the use of imageable nanomaterials contributes to increasing the sensitivity and specificity of diagnostic and therapeutic strategies.

## 2 The Characteristic of Imageable Nanomaterials

**FIGURE 1:**
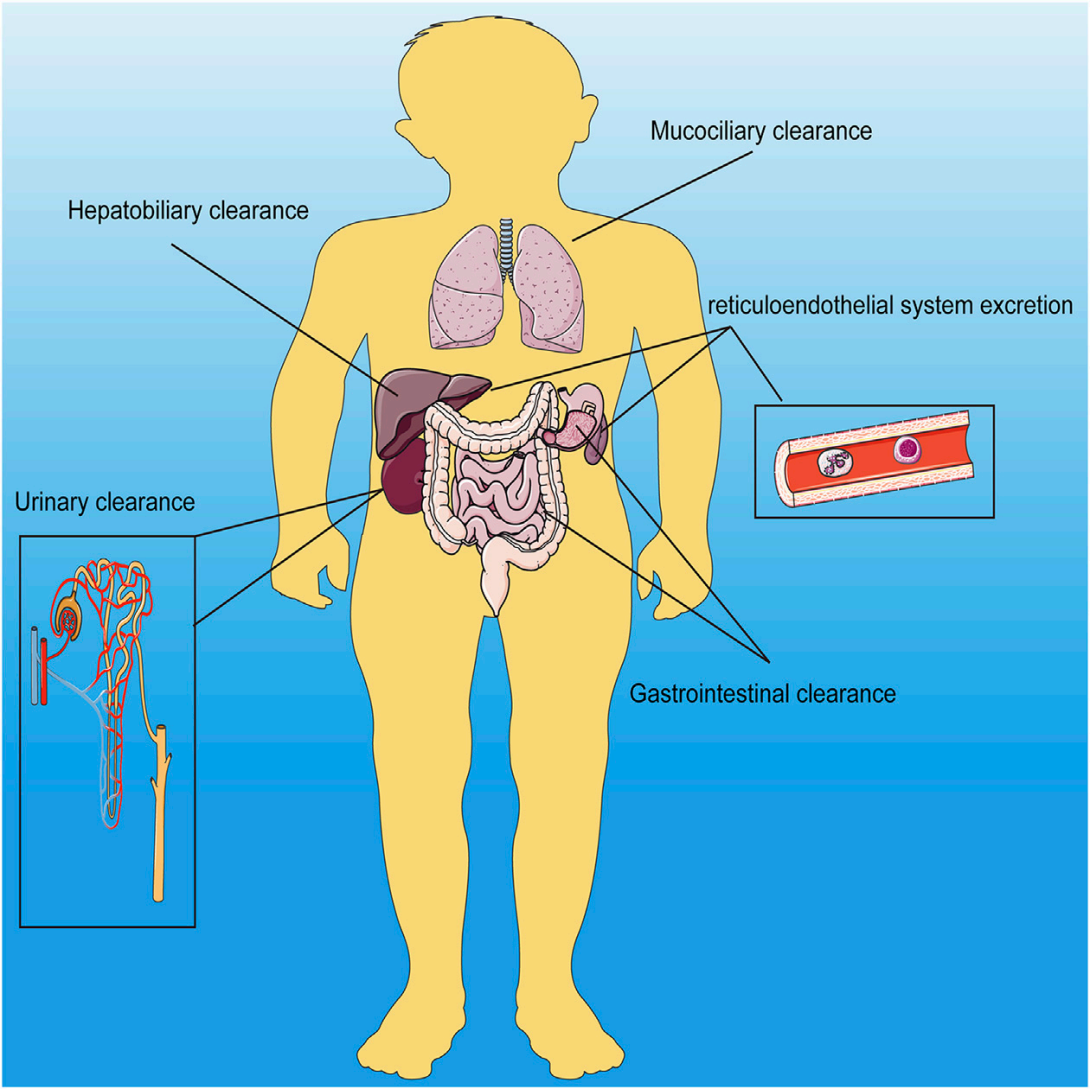
Schematic diagram of the Q12 main metabolic pathways of inorganic nanomaterials in the body.

Nanomaterials refer to nanoscale materials. Due to their small size, nanomaterials have different properties from traditional materials, such as ultrasound, electromagnetics and fluorescence. Nanomaterials were classified into organic, inorganic and hybrid nanomaterials. Inorganic nanomaterials contains silica, black phosphorus, metallic, metal oxide-based nanomaterials, transition metal dichalcogenide, metal carbide, nitride, or carbonitride, calcium, layered double hydroxide, metal–organic framework/nanoscale coordination polymer, self-assembled inorganic nanomaterials; and other biodegradable inorganic nanomaterials (Wang X. et al., 2021). And the well-known liposomes are typical organic nanoparticles (Chin et al., 2017). And nanomaterials of every character can be further classified according to their imaging properties. For example, magnetic nanomaterials can be further classified into magnetic resonance, magnetic particle, magneto-motive and electrical impedance imageable nanomaterials (Alsharif et al., 2020). And the main research branch of PAI includes photoacoustic tomography (PAT), photoacoustic microscopy (PAM) and intravascular photoacoustic imaging (IVPAI) (Attia et al., 2019). Nanomaterials can be composed of many different materials, such as manganese dioxide, calcium carbonate, iron oxide and so on. The clearance of nanomaterials in the body is mainly dependent on hepatobiliary, gastrointestinal, mucociliary, urinary and reticuloendothelial system excretion (Figure 1). Whereas the specific removal method depends on the diameter of the nanomaterial and its ability to aggregate (Wang X. et al., 2021). Traditional imaging technology provides more anatomical information rather than molecular and cellular level information. Recently, with the deepening of research and the understanding of diseases, researchers have made specific modifications on nanomaterials to adapt to the microenvironmental physical and chemical properties of the lesional areas and provide imaging information *via* imaging technology. To distinguish the imaging signal between the normal and lesional area, the imaging properties of nanomaterials have been specifically improved, that is, only the lesion area has imaging properties. The lesion area has different physical and chemical properties from physiological conditions, such as pH, redox and enzyme products. These abnormal properties are used to simulate the imaging signal switch of nanomaterials (Table 1). At present, the nanomaterials are mostly degraded or cleavage when they were activated by pH, redox and enzyme products, and at the same time they were imageable. All inorganic, organic, and hybrid nanomaterials were applied in this strategy (Rosenkrans et al., 2021). Redox products generally include reactive oxygen species (ROS) and glutathione (GSH). High levels of ROS can be detected in most cancers and macrophages, therefore, nanomaterials loading with ROS probe can be imageable in cancers and macrophages-related diseases (Zhang L. et al., 2019). At the same time, some external stimuli can also activate the imaging properties of nanomaterials, such as giving infrared radiation, X-ray and electromagnetic fields outside the body (Tsai and Hamblin, 2017; Pfeiffer et al., 2020; Huang et al., 2021).

**TABLE 1:** Internally responsive nanomaterials for imaging (some examples listed).

Stimulator	Activation	Nanomaterials composition	Imaging performance	Application
PH	Degradation	Octapod-shaped hollow porous MnO (HPMO) NPs loaded with various cargo (Cargo@HPMO), such as camptothecin (CPT) or Rhodamine 123 (Rh123) Wei et al. (2019)	MR and FL imaging	tumor
		MnO@AuNCs: porous gold nanocluster decorated MnO nanocomposites Liu Y et al. (2018)	CT and PA imaging	
		Ce6(Mn)@CaCO3-PEG NPs: The CaCO_3_ NPs were prepared using a gas diffusion reaction under vacuum where ammonia bicarbonate (NH4HCO_3_) was used to precipitate Ca^2+^ ions in an ethanol solution, resulting in CaCO_3_ formation. Chlorine e6 (Ce6) and MnCl_2_ were loaded into the NPs by dissolving them in the ethanol solution and then PEGylated under sonication Dong et al. (2018)	high T1 signal in MR imaging	tumor
	Amine Protonation	PEG-GMF-PPy NPs: PEGylated-gadolinium metallofullerene-polypyrrole Wang et al. (2018)	MR and PA imaging	tumor
		pH-responsive piperazine ring and perylenediimide (PPDI) NPs Li et al. (2020)	PA and FL imaging	tumor
		Gd-chelated Ce6 conjugated to the ultra-pH-responsive diblock copolymer poly (ethylene glycol)-block-poly (diisopropanol amino ethylmethacrylate cohydroxyl methacrylate (PDPA) complex Wang et al. (2016)	MR and FL imaging	
		Small-sized iron oxide nanoparticles (ESIONs) self-assembled with two ligands containing Ce6 and imidazole Ling et al. (2014)	MR and FL imaging	tumor
	Incorporating an Acid Liable Group	^DA^TAT-NPs: TAT peptide polymeric NPs loaded with Ce6 and Gd^3+^, where 2,3-dimethylmaleic anhydride (DA) was conjugated to the TAT via lysine residues Gao M et al. (2017)	MR and FL imaging	
		AuNPs-CKL-FA: gold nanoparticles (AuNPs) conjugated *via* a ketal linker to a NIR fluorophore (Cy5.5) and decorated with FA to enable active targeting of the folate receptor Tang et al. (2019)	CT and FL imaging	subcutaneous HeLa tumors
		D-Au@Gd&RGD: cyclic arginineglycine-aspartic acid peptide (cRGD), rhodamine (Rh-S) and fluorescein (Flu-S) derivatives were decorated in gold nanoparticles Yu et al. (2020)	MR and FL imaging	U87 tumor-bearing mice
		UCNP@GA-Fe^III^: upconversion luminescence nanoparticles (UCNPs) as the core and an iron (Fe^3+^)/gallic acid (GA) complex as the shell Zhang P et al. (2019)	sustained T1-contrast enhancement in MR imaging	tumor
Redox Potential	GSH	Dihydrolipoic acid-modified superparamagnetic iron oxide nanoparticles (IONPs) were used as the core and conjugated with FA and STAT3 inhibitor-functionalized CdS:Mn/ZnS quantum dots (QDs) (MMCNP) Mitra et al. (2012)	MR and FL imaging	
		HSA-Ce6-Mn^2+^ NAs: human serum albumin nanoassemblies cross-linked with GSH and then loaded with Ce6 *via* hydrophobic interactions and later chelated with Mn^2+^ Hu et al. (2016)	MR and PA imaging	tumors in murine models
		A probe containing a Gd^3+^ chelate, ^19^F moiety, and a disulfide-capped amino-oxyluciferin fluorophore Zheng et al. (2016)	MR and FL imaging	
	Reactive Oxygen Species	PBMn-52: biodegradable Prussian blue (PB)/MnO2 hybrid nanocrystals Peng et al. (2017)	MR and PA imaging	
		Gold nanoparticles (AuNPs) were loaded into hybrid polyphosphazene derived polymer nanogels formed through ionic interactions (PPB NPs) Bouche et al. (2019)	CT and PA imaging	
Enzymes	Matrix Metalloproteinases	IONP core–silica shell NPs that were decorated with the same MMP substrate (GPLGVRG) (PCM-CS) Cha et al. (2011)	MR and FL imaging	tumor
		Tumor-targeted and MMP-2 activatable nanoprobe (TMAN): Gd/CuS nanodisks encapsulated into micelles using DSPE-PEG2000, and then functionalized with an α_v_β_3_ tumor-targeting group (cRGDSH) and a Cy5.5- and QSY21-labeled MMP-2 cleavable peptide substrate ((QSY21)-GGPLGVRGK(Cy5.5)-SH Shi et al. (2019)	MR and FL imaging	mice bearing subcutaneous gastric cancer tumors
		ACPP dendrimer (ACPPD): gelatinase-activatable cell-penetrating peptides (ACPP) conjugated with multiple Cy5 and/or gadolinium moieties Chen et al. (2017)	MR and FL imaging	ischemic stroke
	Serine Proteases	ICG/DOX@Gel-CuS NMs: core–satellite NPs were made of gelatin (Gel) NPs loaded with indocyanine green (ICG) and doxorubicin (DOX) that were then coated with PEGylated copper sulfide (CuS) NPs Li X et al. (2019)	FL and PA imaging	real-time monitoring of drug release
		Polydopamine-coated gold nanostars (GNS@PDA) conjugated with Cy7-labeled FAPcleavable peptide (Cy7-KTSGPNQC) and chelated with Fe^3+^ Han et al. (2019)	MR, CT and PA imaging	tumor
		TAP-SiO2@AuNPs: thrombin-activatable fluorescent peptide (TAP) incorporated silica-coated gold nanoparticles Kwon et al. (2018)	FL and CT imaging	discriminating the thrombotic lesion
	Caspase	1-RGD: caspase-3 responsive probe Wang Y et al. (2019)	PA and FL imaging	mice with U87MG subcutaneous tumors
		Caspase probe (CP1) combined a Gd^3+^-chelate, a tetraphenylethylene unit for aggregation-induced emission luminogen (AIEgen), and a caspase-3/7 cleavable substrate (DEVD peptide) Li H et al. (2019)	FL and MR imaging	
	Other Enzymes	Probe was constructed by a prequenched fluorophore (merocyanine) capped with an alkaline phosphatase (ALP) cleavable phosphate group with a Gd-DOTA chelate and a hydrophobic dipeptide Phe-Phe linker for selfassembly (P-CyFF-Gd) Han et al. (2019)	FL and MR imaging	Surgical resection of tumors
		PFOB@IR825-HA-Cy5.5: Cy5.5, IR825 and perfluorooctylbromide (PFOB) were conjugated with Hyaluronic acid (HA) Liang et al. (2017)	PA and CT imaging	HT-29 (CD 44 positive) tumor xenograft model

To further improve the imaging performance, researchers successfully combined two or more imaging modes, called multimodal imaging. For example, the use of multimodal imaging could not only feel the high acid and high ROS levels in the tumor microenvironment but also synergistically enhance the T1-weighted MR contrast of Mn^2+^ (Liang et al., 2018). And Fe_3_O_4_/Gd_2_O_3_ nanocubes had been demonstrated to possess both T1-and T2-weighted imaging properties (Qin et al., 2020). Both Gd_2_O_3_ and NaHoF_4_ had the dual imaging properties of T1-weighted MRI and CT (Ni et al., 2016; Kuang et al., 2020). While there are limited natural nanomaterials that can be used for multimodal imaging. After exploration, nanomaterials are modified or mixed to have the characteristics of multimodal imaging. In the studies, the MnO_2_ core was wrapped with Gd^3+^ coated nanomaterials, or the MnO_2_ core was conjugated with functional groups to increase the Mn^2+^ concentration, which enhanced the T1 imaging (Sun et al., 2018). Nanomaterials coupling with ^64^Cu and Gd^3+^ realized the dual imaging of PET and MRI (Neumann et al., 2020). And microbubbles with low-frequency ultrasound response had been demonstrated to have dual imaging properties of fluorescence and PAI upon triggering in tumor-bearing mice (Huynh et al., 2015). Multimodal imaging not only provided better anatomical information but also real-time molecular and cellular level information. However, it also requires optimization of the combination and modification of nanomaterials to meet the needs of multimodal imaging.

## 3 The Advantage of the Imageable Nanomaterials

Nanomaterials with imaging functions were detected by imaging technology. The combination of nanomaterials and imaging technology breaks the limited information provided by traditional nanomaterials or imaging technology, so that the biological processes can be monitored in real-time while providing more information about diagnosis and treatment of diseases. We will clarify the advantages of the combination of nanomaterials and imaging technology from the following two aspects.

### 3.1 Imaging Technology Tracks Nanomaterials *in vivo* and in Real-Time

At present, the research on imageable nanomaterials is mostly concentrated in cancers. Imageable nanomaterials carrying drugs can trace not only the location and size of the cancer but the half-life of the drug and the targeting properties of nanomaterials. For example, nanoparticles carrying Fe_3_O_4_ and doxorubicin under the external alternating magnetic field made the local temperature reach above 42°C and doxorubicin was released at the same time, which significantly inhibited tumor growth and enhanced the T2 contrast for imaging-guided delivery (Thirunavukkarasu et al., 2018). At the same time, nanomaterials with the magnetic, optical and thermal response and imaging characteristics activated the magnetic, optical and thermal properties of the material itself after receiving stimuli to provide the imaging information of cancer sites and achieve the treatment of cancers. Nanoparticles activated by near-infrared laser irradiation could not only have the effect of chemotherapy/photothermal synergistic anti-tumor efficacy but also have the dual-mode imaging characteristics of PA and ultrasound imaging (Liu F. et al., 2018). In addition, radiolabeling nanomaterials such as ^18^F were subjected to PET imaging for monitoring the metabolism of cancer tissues (Norregaard et al., 2017). Nanomaterials with dual imaging and diagnostic properties achieved the intraoperative diagnosis and precise imaging-guided surgery (IGS). Superparamagnetic iron oxide nanoparticles enabled the precise localization of sentinel lymph nodes guided by MR (Rubio et al., 2015). The CH1055-PEG carrying the NIR-II fluorophore showed great potential in intraoperative lymph node localization with a higher signal-to-background ratio (Antaris et al., 2017). Further, tumor boundary and minimal residual disease could be visualized by imaging techniques to perform the precise IGS (Wang C. et al., 2019). Another application of imaging technology to trace nanomaterials is stem cell tracing. As we all know, stem cells play an important role in the fields of gene therapy and drug research. At present, most of the reported techniques for tracing stem cells with nanomaterials are MRI and PAI (Liu et al., 2011; Duan et al., 2017; Hsu et al., 2018; Quang et al., 2018; Ali et al., 2020). Mesoporous silica nanoparticles loaded with cobalt protoporphyrin IX (CoPP) and ^125^I can track mesenchymal stem cells (MSC) of cerebral ischemia models at multiple time points through SPECT and PAI (Yao et al., 2020). Studies have shown the gold-coated multifunctional nanoparticles tracked the homing degree of bone marrow-derived human MSC in a mouse model of glioma under MRI and PAI (Qiao et al., 2018). Further, Tseng et al. (2010) found the gadolinium hexanedione nanoparticles (GdH-NPs) functioned as a contrast MRI agent for stem cell tracking. Bioluminescence imaging tracking MSC was used to optimize the dose and route of MSCs in mice with acute liver injury (Li et al., 2015).

In addition, imaging technology could guide nanomaterials to import into the target area. One application example is that nanomaterials with dual characteristics of imaging and treatment reach specific areas under the guidance of imaging technology. Under the guidance of imaging technology, MSCs were implanted not only to monitor bone defect and injury therapy (Ryu et al., 2020) but also to improve the therapeutic effect of heart stem cells (Jokerst et al., 2013). Under the guidance of ultrasound or MRI, mesoporous silica nanoparticles carrying drugs that promote cell survival are accurately implanted into the area around the infarct and avoid the most severely necrotic tissue (Kempen et al., 2015). Nanomaterials carrying miRNAs or drugs were accurately delivered to cancer sites under the guidance of imaging technologies such as ultrasound to achieve precisely targeted therapy of cancers (Wang et al., 2015; Zhao et al., 2018). Nanomaterials carrying radionuclides under the guidance of PET/SPECT could not only show the morphological or metabolic abnormalities in bone tissue but monitor the response to bone metastases (Farzin et al., 2019).

### 3.2 The Imageable Nanomaterials Improve the Specificity and Sensitivity of Diseases’ Diagnosis and Treatment

**FIGURE 2:**
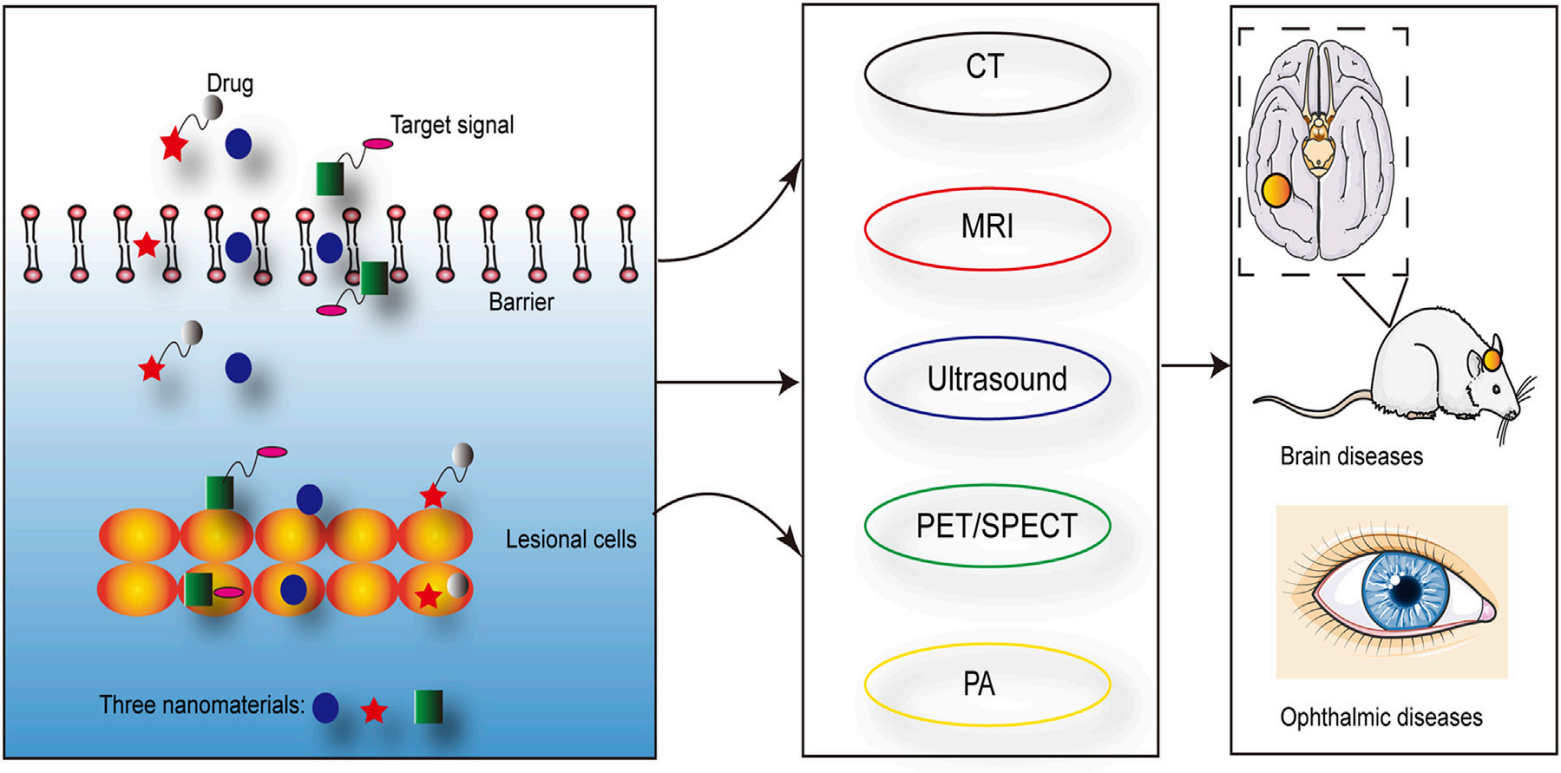
Nanomaterials could effectively pass through barriers in vivo and achieve the targeting of lesional cells, which were visualized by the imaging techniques.

Nanomaterials have the characteristics such as ultrasound, optics and magnetism, which can be used to diagnose and treat diseases. The long blood circulation time and tissue specificity of nanomaterials improved the specificity and sensitivity of imaging, which in turn contributed to the early diagnosis of diseases (Truffi et al., 2016; Xiong et al., 2019; Jin et al., 2020). And multiple imaging techniques have been demonstrated to improve the accuracy of tumor metastasis rate in sentinel lymph node biopsy, such as PAI (Stoffels et al., 2015). Imageable nanomaterials loading with drugs or generating oxygen may enhanced the anti-cancer efficacy (Yang et al., 2019). For example, the presence of nanomaterials reduced the ultrasound intensity of the therapeutic effect and specifically enhanced the imaging ability of the lesion area (Sviridov et al., 2019). Magnetic nanomaterials with MR imaging capabilities could enhance the efficacy of liver chemoembolization (Pouponneau et al., 2014). Studies have shown that magnetic nanomaterials were used for hyperthermia under the stimulation of an external magnetic field (Kazantseva et al., 2021). At the same time, the specific modification of nanomaterials not only improved the specificity and effect of treatment but also reduced the damage to surrounding tissues (Patra et al., 2018). In addition, magnetic nanomaterials also effectively passed through the barriers in the brain and eyes, which provided an application basis for the diagnosis and treatment of brain and ophthalmic diseases (Kumar and Mohammad, 2011; Qiao et al., 2018; Yao et al., 2020; Gao et al., 2021; Liu et al., 2021) (Figure 2). Nanosized drug-eluting beads combined with transcatheter arterial chemoembolization could improve the therapeutic effect of liver cancer, which was evaluated by ultrasound (Zhao et al., 2020). The nanoparticle-coupled microbubble complex had targeting and ultrasound imaging functions. It could target liver cancer lesions under ultrasound guidance and simultaneously released chemotherapeutic drugs, which effectively killed tumor cells (Kim et al., 2021).

In addition, the application of multimodal imaging also enables dynamic monitoring of diseases’ diagnosis and treatment. Nanomaterials carrying radionuclides not only had the characteristics of SPECT/PET imaging, diagnosis and treatment, but the other groups such as chemotherapy drugs carried by the imageable nanomaterials also had the dual characteristics of CT/MR/PA/OI/ultrasound imaging and treatment (Ge et al., 2020). The application of multimodal imaging combined the advantages of a single imaging method and made up for the shortcomings of a single imaging method, such as the off-target effect (Hasan et al., 2018). These nanomaterials not only provided the anatomical structure information and pathophysiological features of the disease areas but also improved the sensitivity and resolution of imaging (Wu and Shu, 2018). The ^99m^Tc-labeled ferroferric oxide nanoparticles had dual imaging characteristics of PET/SPECT and MRI (Felber and Alberto, 2015). In addition, the peptides that specifically recognized and targeted tumors carried by the nanoparticles could not only prevent them from being taken up in the blood circulation but also the disulfide bond triggered by GSH breaking after arriving at the tumor site, which in turn exposed the peptides that could bind to the tumor cell-specific receptor αvβ3 (Trajkovic-Arsic et al., 2014). Further, the nanoparticles have the characteristic of aggregation in the tumor microenvironment (Gao Z. et al., 2017). Since optical imaging could directly monitor the molecular level to track the dynamic process of metabolism *in vivo*, it has been widely used in various biological studies. Therefore, giving nanomaterials to optical and other imaging properties realized the dynamic monitoring of molecules and overcame the shortcomings of optical imaging that are limited by the depth of the tissue (Wang Z. a. et al., 2021; Ding et al., 2021; Luo et al., 2021; Xu et al., 2021). Coating or mounting near-infrared fluorescent pigments, targeting markers, and radioactive elements on nanomaterials could achieve PET/near-infrared dual-modal imaging of tumor-associated macrophages in mouse (Kwon et al., 2021). Zhang et al. achieved targeting and sustained drug release *in vivo* using mesoporous silicon nanoparticles with ultrasound and optical imaging (Qi et al., 2019).

## 4 The Biomedical Applications of Imageable Nanomaterials

With the advancement of nanotechnology, nanomaterials have been more and more modified and improved, such as multimodal imaging, targeted drug delivery, etc., and have been applied to basic research and preclinical research of human diseases. Currently, research on imageable nanomaterials has focused on cancers and/or MSC. Nanomaterials are modified with targeted molecules to enhance the efficacy of targeted drugs and to track changes in biological activities such as metabolism at the target site; drugs are encapsulated in nanomaterials to reduce the uptake or clearance at non-target sites; the sustained release properties of nanomaterials can maintain the blood concentration of the target site and enhance the efficacy of short half-life drugs. The characteristics of multimodal imaging allowed the off-target effects of nanomaterials to be monitored, and overcame the shortcomings of a single imaging method, making the experimental results more realistic and credible. We summarized the applications of the imageable nanomaterials in cancers and non-cancer diseases in the following.

### 4.1 Cancers

**FIGURE 3:**
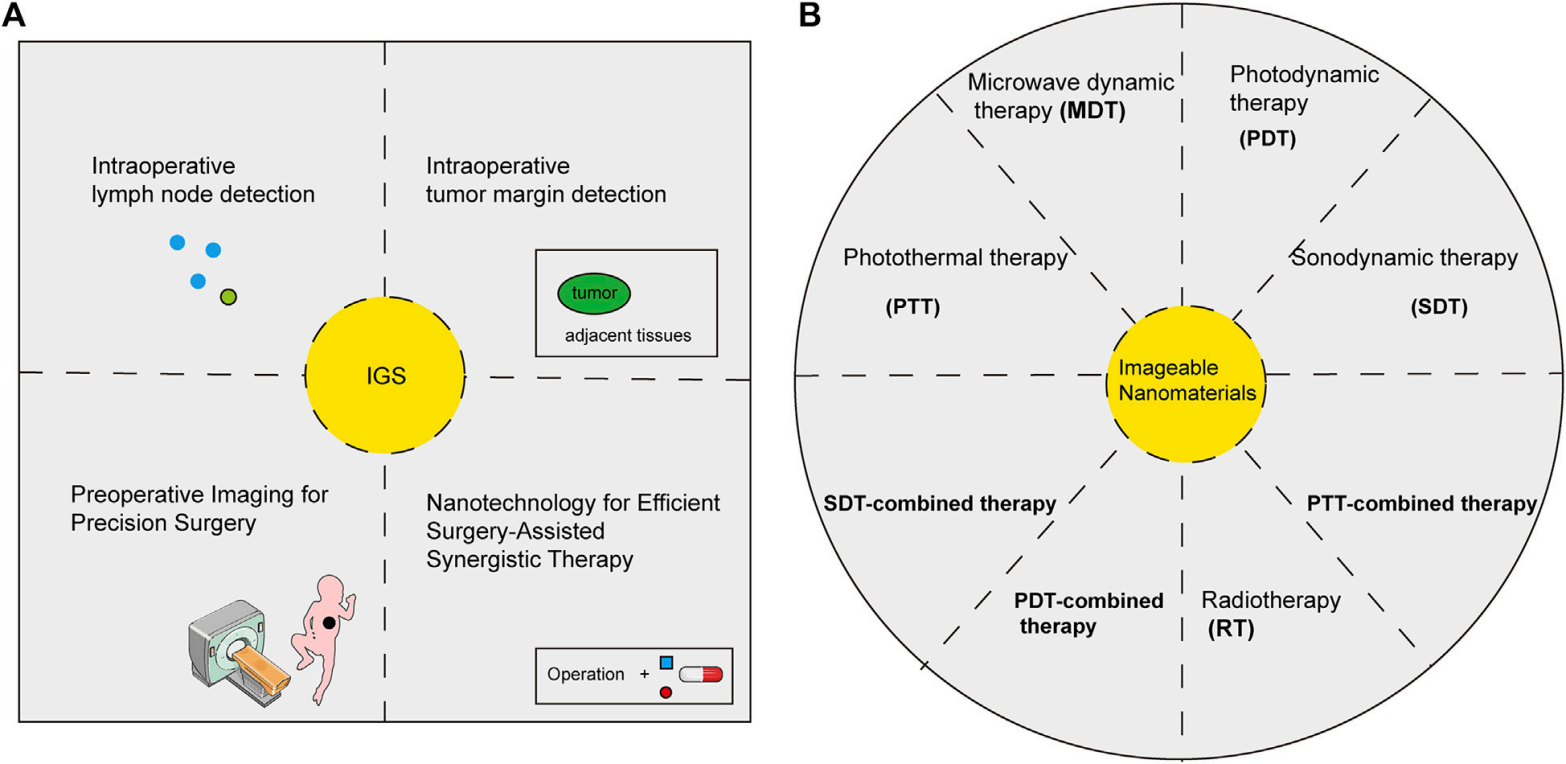
Biomedical applications of imageable nanomaterials in cancers. Imageable nanomaterials were applied in imaging-guided surgery (IGS) (A) and theranostics (B) of cancers.

Currently, the applications of imageable nanomaterials are mainly focused on the diagnosis and treatment of cancers (Wang et al., 2019a; Phuong et al., 2020) (Figure 3). Nanomaterials with imaging properties tend to have high sensitivity, which has important potential in detecting the minimal lesions of early-stage cancer. Radiolabeled nanomaterials showed great advantages in the early diagnosis of cancers due to their deep penetration and high sensitivity (Ge et al., 2020). Nanomaterials with fluorescent imaging properties were widely used in molecular labeling, which displayed molecular dynamics and tracked specific biomarkers, indicating a great potential in the early diagnosis of cancers (Jin et al., 2020). In addition, the surface modification properties such as high-temperature, acid and alkali resistance of nanomaterials made an early diagnosis of cancers in some special parts such as the stomach possible (Truffi et al., 2016). The zwitterion-modified nanomaterials could form water layers on their surfaces to protect the nanomaterials from contamination by other non-specific proteins and prolong the blood circulation time of the nanomaterials (Li G. et al., 2021). The modification of specific tumor-related ligands could not only improve the target of tumors but also enhance its ability to aggregate and image in tumor sites, which contributed to the early diagnosis of tumors (Xiong et al., 2019). Nanomaterials-labeled MSCs with imaging properties aided in the diagnosis of lung metastases (Loebinger et al., 2009), osteosarcoma (Duchi et al., 2013) and brain cancers (Kim et al., 2016). The development and application of multimodal imaging nanomaterials had further increased the sensitivity and specificity of early diagnosis of cancer (Malik et al., 2020).

The advantage of imageable nanomaterials is that imaging can not only provide more accurate information on tumor sites but also provide information on drugs and their efficacy. Using the photothermal conversion properties of nanomaterials, photothermal ablation of tumor cells could be performed to enhance the therapeutic effect of tumors. Nanomaterials with photothermal conversion properties such as Au could monitor the curative effect in real-time with the help of imaging technology (Chen et al., 2014; Xiao et al., 2014). Nanomaterials with photosensitizer and imaging properties generated ROS under the action of optical to kill tumor cells. This therapy was called photodynamic therapy, and the efficacy was monitored in real-time. At the same time, the nanomaterials were specially modified to trigger the production of endogenous oxygen, which enhanced the effect of photodynamic therapy (Zhu et al., 2018; Liang et al., 2019). Drug-loaded nanomaterials with imaging capabilities could not only monitor the therapeutic effect of tumors, but also the provide targeting, pharmacokinetics, and sustained-release properties of drug-loaded nanomaterials (Truffi et al., 2016; Zhang et al., 2020; Wang and Niu, 2021). Zwitterion-modified nanomaterials promoted drug aggregation and targeting in tumor sites (Li G. et al., 2021). Radiolabeling nanomaterials were imaged with the help of SPECT/PET, which provided information on the treatment of tumors (Ge et al., 2020). The application of multimodal imaging nanomaterials greatly reduced off-target effects, combined the advantages of multiple imaging modalities and reduced the limitations of a single imaging modality (Liao et al., 2014; Gulzar et al., 2021). The current focus is on how to successfully translate the results of basic research into clinical applications. And it may take a long time to explore.

### 4.2 Other Diseases

Most diseases in the body except cancers involve the changes of ROS and its related pathways. Therefore, ROS-activated nanomaterials show great potential in the diagnosis and treatment of these diseases, such as chronic diseases, acute injuries and infectious diseases. Researches showed that nanomaterials labeled MSC with imaging properties showed great potential in repairing complete spinal cord injury (Guo et al., 2019), joint defects (Kaggie et al., 2020) and acute liver injury (Li et al., 2015), enhancing the phagocytic activity of macrophages in the acute respiratory distress syndrome (Jackson et al., 2016) and skin regeneration (Xiao et al., 2020), improving the cardiac function after myocardial infarction (Gong et al., 2021) and contributing to the diagnosis and/or treatment of neurodegenerative diseases including Alzheimer’s disease, Parkinson’s disease, and Huntington’s disease (Perets et al., 2019), neuropsychiatric disorders (Betzer et al., 2014), cerebrovascular disease (Kempen et al., 2015; Yao et al., 2020), silica-induced pulmonary fibrosis (Huang et al., 2020), osteoporosis (Li M. et al., 2021), acute liver failure (Cai et al., 2020), liver fibrosis (Lai et al., 2016), traumatic brain injury (Mishra et al., 2020). In addition, current research has confirmed the use of iron oxide or gold nanoparticles, polymeric nanoparticles, liposomes, and micelles for atherosclerosis imaging (Chen J. et al., 2021). Furthermore, macrophage-specific molecularly upconverted nanoparticles can image atherosclerotic plaques under dual optics/MRI (Qiao et al., 2017). Nanomaterials with MR imaging properties have demonstrated promising diagnostic performance in neurodegenerative diseases (Cui et al., 2021). Nanomaterials based on the polydopamine and imaging properties showed great therapeutic potential in inflammation, diabetes, rheumatoid arthritis and neurodegenerative diseases (Li H. et al., 2021; Hosseinikhah et al., 2021). And there also had research on imageable nanomaterials in inflammatory and infectious diseases. For example, gold nanomaterials have shown great application potential in macrophage-mediated inflammation imaging and therapy (Chen W. et al., 2021). And MRI-guided sonodynamic therapy has important implications for drug-resistant deep bacterial infections (Wang D. et al., 2021). More evidence will follow on the potential of imageable nanomaterials in non-tumor diseases.

## 5 Conclusion and Outlook

The progress of nanotechnology has made nanomaterials experience the progress from simple inorganic nanomaterials to the current organic and hybrid nanomaterials and the progress from single modification to the coexistence of multiple modifications. These advances have made significant contributions to the diagnosis, individualized treatment of diseases and the enhancement of efficacy, while reducing the harm caused by interventional methods, enabling real-time monitoring of diseases’ diagnosis and treatment *in vitro*. However, some challenges remain for the further development of imageable nanomaterials. The first is the stability of the modified group. The imaging groups that target and sensory stimuli are covalently or non-covalently linked to nanomaterials, they may break due to physical stress or the physical and chemical environment at non-target sites, resulting in false positives, the imaging at this time does not provide information about the lesion site. The second is the stability of nanomaterials to sense stimuli. Nanomaterials are often imaged as they are degraded. However, the degradation of nanomaterials is usually completed in an instant, which requires high capture conditions for imaging. Therefore, we need to design more accurate stimulation sensitivity or use porous surface coatings to increase the stable imaging performance of nanomaterials. The third is that the imageable nanomaterials mostly contain heavy metals. When the nanomaterials are degraded, the heavy metals are taken up in the body and not smoothly removed from the body, which is a problem that needs to be solved at present. Although not all degradation products are harmful. For example, iron ions generated by the degradation of iron oxide can be used to replenish intracellular iron, which is very beneficial for iron deficiency diseases. The last is that most of the research on imageable nanomaterials remains in preclinical research, and there is still a long way to go before its clinical translation. Therefore, the application of imageable nanomaterials in diseases needs further exploration. With the advancement of nanotechnology and biotechnology, we believe that the clinical application of imageable nanomaterials will become more and more extensive.
